# Infections of Cardiac Implantable Electronic Devices

**DOI:** 10.1016/s0972-6292(16)30814-2

**Published:** 2014-12-15

**Authors:** SP Abhilash, Narayanan Namboodiri

**Affiliations:** Sree Chitra Tirunal Institute for Medical Sciences and Technology, Trivandrum, Kerala, India

**Keywords:** Cardiac Implantable Electronic Devices, Infections

The use of cardiac implantable electronic devices (CIEDs), which includes permanent pacemakers and implantable cardioverter-defibrillators, has greatly increased over the past two decades. Unfortunately, along with the rising use of cardiac medical devices, there has been a disproportional increase in the number of infections of such devices [1]. The rates of infection of CIED range from 0.1% to 4%, with an average rate of 1% [2-4]. A recent analysis showed that infection rate grew from 1.61% in 1993 to 2.41% in 2008, possibly due to two factors: aging of population and increased use of more complex devices [5]. The most important risk factors for infection are re-intervention for hematoma evacuation or lead displacement, device replacement increasing with aging of population, and use of dual and triple chamber devices that increased in the last years.

*Staphylococcus epidermidis* and *Staphylococcus aureus*, account for 70-90% of all CIED infections, and have been studied extensively [6,7]. Non-staphylococcal organisms, which cause 10 - 30% of all CIED infections, are so diverse that they have been studied only infrequently [8]. The microbiological diversity of non-staphylococcal infections of CIED is rather extensive, including other Gram-positive bacteria, Gram-negative bacteria, atypical bacteria like *Nocardia* species, fungi like *Candida* and *Aspergillus* species, and mycobacterial organisms.

The majority of CIED infections reportedly occur within 3 months after CIED insertion [6]. Reasons for the higher rate of infection during the early period after CIED implantation include procedural contamination and the lack of formation of fibrocollagenous tissue around the leads until a few months after placement, thereby making them more prone to microbiological seeding. In contrast, non-staphylococcal infection occurs rather late, usually 2-3 years after implantation. Compared to staphylococci, non-staphylococcal organisms appear to be less virulent and result in more protracted clinical course. They are generally less lethal with a low infection-related mortality of 4% compared with 9% for *S. aureus* [9].

The probability of CIED infection due to secondary seeding of the cardiac device in patients with *Staphylococcus* bacteremia is approximately 40% [10]. Previously, non-staphylococcal bacteremia was thought not to cause secondary seeding of the device. But subsequent studies [8] showed that this was indeed a misconception. Therefore, regardless of the nature of the microorganism, one must always remain cautious of the potential for secondary seeding of the CIED in any patient with bacteremia or a distant bodily site infection.

A few cases of CIED infections associated with mycobacterial species like *Mycobacterium fortuitum*, and *Mycobacterium avium complex* have been reported in literature. CIED infections with M. tuberculosis are very rare. In this issue of journal, Kumar A et al report 3 cases of CIED infection due to *M. tuberculosis* [11]. *M. tuberculosis* infection of CIED pocket generally follows an indolent course and is devoid of constitutional symptoms. Diagnosis is often missed or delayed since routine investigations may not help in identifying the organism or the diagnosis is considered only late in the course of the illness. It is important for clinicians to consider tuberculosis as an etiology of any CIED infection. It is interesting to note that two out of three cases reported were managed conservatively with antituberculous drugs and did not require device explantation.

In patients with suspected CIED infection, adequate Gram-positive and Gram-negative antibacterial coverage should be administered until microbiological data become available. When Gram stain and routine cultures are not contributory, one should consider unusual organisms like nocardia, fungi or mycobacterium species. Considering these atypical organisms for CIED infection is especially relevant in a patient who is immunocompromised or has diabetes mellitus.
